# Treatment optimization for recurrent hepatocellular carcinoma: Repeat hepatic resection versus radiofrequency ablation

**DOI:** 10.1002/cam4.2951

**Published:** 2020-02-28

**Authors:** Liang‐He Lu, Jie Mei, Anna Kan, Yi‐Hong Ling, Shao‐Hua Li, Wei Wei, Min‐Shan Chen, Yong‐Fa Zhang, Rong‐Ping Guo

**Affiliations:** ^1^ Department of Hepatobiliary Oncology Sun Yat‐sen University Cancer Center Guangzhou China; ^2^ State Key Laboratory of Oncology in South China Guangzhou China; ^3^ Collaborative Innovation Center for Cancer Medicine Guangzhou China; ^4^ Department of Pathology Sun Yat‐sen University Cancer Center Shanghai China; ^5^ Department of Hepatic Surgery Fudan University Shanghai Cancer Center Shanghai China; ^6^ Department of Oncology Shanghai Medical College Fudan University Shanghai China

**Keywords:** postrecurrence survival, radiofrequency ablation, recurrent hepatocellular carcinoma, repeat hepatic resection

## Abstract

**Background and aims:**

The optimal treatment strategy for recurrent hepatocellular carcinoma (HCC) remains unclear. Therefore, we aimed to compare the outcomes of repeat hepatic resection (RHR) and radiofrequency ablation (RFA) for recurrent HCC.

**Method:**

From December 2004 to December 2015, 138 patients who underwent RHR and 194 patients who underwent RFA were enrolled. Propensity score matching (PSM) was performed to establish 1:1 RHR‐RFA group matching. Clinical outcomes were compared before and after matching.

**Results:**

Before matching, the 1‐, 3‐, and 5‐year postrecurrence survival (PRS) rates were 91.8%, 82.0%, and 72.9% for the RHR group (n = 138) and 94.4%, 75.4%, and 61.7% for the RFA group (n = 194), respectively (*P* = .380). After matching, the PRS rates at 1, 3, and 5 years were 90.5%, 81.5%, and 71.8% for the RHR group (n = 120) and 91.0%, 61.0%, and 41.7% for the RFA group (n = 120), respectively (*P* = .002). In the subgroup analysis, the PRS rates for the RHR group were better than those for the RFA group for patients who relapsed within 2 years (*P* = .004) or patients with primary tumor burden beyond the Milan criteria (*P* = .004). Multivariate analysis showed that treatment allocation was identified as an independent prognostic factor for PRS.

**Conclusion:**

Compared with RFA, RHR provided a survival advantage for recurrent HCC, especially for patients who relapsed within 2 years and those with primary tumor burden beyond the Milan criteria.

## Graphical abstract

Compared with RFA, repeat hepatic resection provided a survival advantage for recurrent HCC, especially for patients relapsed within 2 years and patients with primary tumor burden beyond Milan criteria.

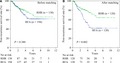

## 1 INTRODUCTION

Hepatocellular carcinoma (HCC) is among the most common cancers and is the leading cause of cancer‐related mortality worldwide.^1^ Although hepatic resection is commonly chosen as a curative treatment for HCC, long‐term outcomes after hepatic resection are not yet satisfactory, as tumor recurrence within the liver occurs in up to 60%‐80% within 5 years.^2^, ^3^, ^4^ Moreover, guidelines for the management of recurrent HCC remain controversial and poorly defined.

Intrahepatic recurrence is the main cause of postoperative death for HCC in clinical practice, and the postrecurrence survival (PRS) of HCC patients is greatly impacted by the characteristics of the recurrent tumor and the corresponding treatment modalities.^5^, ^6^, ^7^ Currently, the available treatment options for recurrent HCC are not particularly different from those for primary HCC. Repeat hepatic resection (RHR) continues to be the conventional option for recurrent HCC with preserved liver function and residual liver volume,^8^, ^9^, ^10^, ^11^ and advances in surgical techniques and perioperative care have resulted in improved safety in RHR. In addition, as a minimally invasive option, radiofrequency ablation (RFA) has emerged as another alternative treatment modality for small HCC.^12^ Several studies have previously recommended RHR when possible in the treatment of recurrent HCC.^9^, ^10^, ^11^ However, conflicting data have also shown that outcomes following RFA are similar to those following RHR for the management of recurrent HCC.^13^, ^14^, ^15^, ^16^ Thus, the outcomes of RHR and RFA for the treatment of recurrent HCC remain unclear and controversial.

In this study, we compared the outcomes of recurrent HCC treated by RHR or RFA using a large cohort. Propensity score matching (PSM) analysis was carried out to minimize the bias that arises from different patient backgrounds.

## 2 MATERIALS AND METHODS

### 2.1 Patients

From December 2004 to December 2015, a total of 1186 HCC patients developed recurrence after initial resection. Among these patients, 144 (12.1%) were amenable to RHR, 203 (17.1%) received RFA, 678 (57.2%) underwent chemoembolization, 76 (6.4%) received sorafenib, and 85 (7.2%) received only supportive treatment. Recurrent HCC was defined as the appearance of a new lesion with radiologic features typical of HCC, as confirmed by two or more imaging modalities. The following criteria were used for patient selection: (a) age between 18 and 75 years; (b) a diagnosis of intrahepatic recurrent HCC after initial curative hepatic resection; (c) no radiologic evidence of macroscopic vascular invasion or extrahepatic metastasis; (d) RHR or RFA performed as the initial treatment for recurrent HCC; and (e) Child‐Pugh class A or B. Thus, six patients in the RHR cohort were excluded, including four patients who received palliative repeated resection and two patients with other malignancies. Nine patients in the RFA cohort were excluded, including five patients older than 75 years and four patients with extrahepatic metastasis. In total, 342 consecutive patients who underwent RHR (n = 138) or RFA (n = 194) were ultimately enrolled. The correction of potential confounding factors that may affect the outcome of these groups was performed with 1:1 PSM. Finally, 120 patients who underwent RHR and 120 patients who underwent RFA as the first treatment for recurrent HCC were enrolled. This study was approved by the Institutional Review Board of our center and conducted according to the ethical guidelines of the 1975 Declaration of Helsinki.

### 2.2 Treatment strategy

Treatment selection for recurrent HCC was decided by our multidisciplinary team. RHR was assigned when there was the possibility for the complete removal of all tumors while retaining a sufficient liver remnant with an expected remnant liver volume of no less than 250 ml/m^2^, as assessed by our multidisciplinary team. Resection was avoided if patients had gross ascites, severe portal hypertension, or inadequate liver remnant. Reasons for assigning RFA instead of RHR included psychological resistance to invasive treatment, refusal of general anesthesia, and an insufficient liver remnant.

### 2.3 RHR procedure

Repeat hepatic resection was performed using previously described techniques.^17^ Intraoperative ultrasound was routinely performed to assess the tumor burden, the liver remnant, and the possibility of a negative resection margin. Pringle's maneuver, with a clamp/unclamp time of 10 min/5 min, was performed if necessary. Anatomic resection was our preferred surgical method. If the liver remnant was inadequate, nonanatomic resection was performed with a negative resection margin.

### 2.4 RFA procedure

Radiofrequency ablation was performed with the use of conscious analgesic sedation and local anesthesia. All procedures were performed percutaneously under real‐time ultrasound guidance.^18^, ^19^ A Cool‐tip RFA system (Radionics) with a 3 cm active tip length was used for ablation. The numbers of overlapping ablations and ablation points were determined by the number and diameter of the tumor with the aim of achieving an ablative margin of at least 0.5 cm in the normal tissue surrounding the tumor. At the end of the procedure, the needle tract was ablated to prevent bleeding and tumor seeding.

### 2.5 Follow‐up

In both groups, dynamic enhanced computed tomography (CT) was conducted 4 weeks after the treatment to evaluate the efficacy of the technique. Thereafter, the patients were followed up once every 3 months for the first 2 years and then once every 6 months after 2 years. At each follow‐up session, blood tests, including serum liver function tests and AFP tests, as well as abdominal contrast material‐enhanced three‐phase dynamic spiral CT or magnetic resonance imaging, if necessary, were carried out. All patients with hepatitis‐related HCC who were prepared for the initial resection of their HCC in our hospital were counseled by a hepatologist for antiviral therapy regardless of the serum HBV DNA result.^20^, ^21^

### 2.6 Statistical analysis

The main end point of this study was PRS, which was defined as the date of recurrence until death or the end of the follow‐up period. PSM was performed to reduce the patient selection bias and confounding variables between the RHR and RFA groups according to age, tumor size at recurrence, tumor number at recurrence, and Child‐Pugh score. A one‐to‐one nearest neighbor matching algorithm was performed with a caliper of 0.1. Comparisons of the RHR and RFA groups were performed using *t* tests for continuous variables and chi‐square or Fisher's exact tests for categorical variables before and after PSM. The PRS curves were calculated by the Kaplan‐Meier method and analyzed with a log‐rank test. All variables that yielded a *P* value of <.05 in the univariate analysis were subjected to a multivariate analysis using Cox proportional hazards models. All tests were two‐sided, and a significant difference was considered when *P* < .05. Statistical analysis was performed using SPSS 24.0 (SPSS Inc).

## 3 RESULTS

The baseline characteristics before and after PSM are summarized in Tables 1 and 2. Before matching, the median follow‐up period after recurrence was 37.6 months for the RHR group and 41.6 months for the RFA group. Compared with those in the RHR group, patients in the RFA group were older (52.9 ± 11.8 years vs 50.1 ± 10.9 years, *P* = .028), more patients relapsed within 2 years after initial hepatic resection (65.5% vs 39.1%, *P* < .001), and exhibited smaller recurrent tumor sizes (1.9 ± 0.9 cm vs 2.8 ± 1.9 cm, *P* < .001). After matching, there was no significant difference between the RHR and RFA groups (Tables 1 and 2). For patients with recurrent HCC who received RHR or RFA, repeated recurrence was observed in 128 patients, including 69 of 120 (57.5%) in the RFA cohort and 59 of 120 (49.2%) patients in the RHR cohort. Among the 128 patients with repeated recurrence, 52 of 69 (75.4%) patients in the RFA cohort and 31 of 59 (52.5%) patients in the RHR cohort developed only intrahepatic recurrence.

**Table 1:** Patient characteristics at the time of recurrence before and after PSM

Variables	Before matching			After matching		
	RFA (n = 194)	RHR (n = 138)	*P*	RFA (n = 120)	RHR (n = 120)	*P*
Age, y	52.9 ± 11.8	50.1 ± 10.9	.028	50.9 ± 11.6	50.3 ± 10.5	.683
Male sex	172 (88.7)	124 (89.9)	.730	104 (86.7)	108 (90.0)	.421
Hepatitis						
HBV	172 (88.7)	126 (91.3)	.725	108 (90.0)	112 (93.3)	.582
HCV	6 (3.1)	3 (2.2)		2 (1.7)	2 (1.7)	
Others	16 (8.2)	9 (6.5)		10 (8.3)	6 (5.0)	
WBC, 10^9^/L	6.4 ± 1.8	6.4 ± 1.8	.773	6.2 ± 1.7	6.3 ± 1.9	.600
PLT, 10^9^/L	166.0 ± 57.3	176.0 ± 64.8	.138	162.2 ± 57.3	172.8 ± 65.5	.187
ALT, U/L	55.0 ± 87.4	54.1 ± 137.8	.942	60.3 ± 108.3	56.0 ± 147.1	.794
ALB, g/L	41.8 ± 4.6	42.5 ± 3.6	.176	42.0 ± 3.6	42.3 ± 3.6	.428
TBIL, μmol/L	15.3 ± 8.9	14.9 ± 5.8	.666	15.1 ± 8.5	15.0 ± 5.9	.911
AFP at recurrence, ng/mL						
>20	71 (36.6)	43 (31.2)	.304	50 (41.7)	45 (37.5)	.509
≤20	123 (63.4)	95 (68.8)		70 (58.3)	75 (62.5)	
Child‐Pugh score at recurrence						
5	184 (94.8)	136 (98.6)	.138	117 (97.5)	119 (99.2)	.313
6	10 (5.2)	2 (1.4)		3 (2.5)	1 (0.8)	
Cirrhosis	134 (69.1)	96 (69.6)	.924	85 (70.8)	86 (71.7)	.887
Time to recurrence						
>2 y	67 (34.5)	84 (60.9)	<.001	58 (48.3)	73 (60.8)	.052
≤2 y	127 (65.5)	54 (39.1)		62 (51.7)	47 (39.2)	
Tumor size at recurrence, cm	1.9 ± 0.9	2.8 ± 1.9	<.001	2.2 ± 1.0	2.4 ± 1.1	.091
Tumor number at recurrence						
Solitary	162 (83.5)	112 (81.2)	.579	106 (88.3)	106 (88.3)	1.000
Multiple	32 (16.5)	26 (18.8)		14 (11.7)	14 (11.7)	

Note

Variables are expressed as the mean ± SD or n (%).

Abbreviations: AFP, α‐fetoprotein; ALB, albumin; ALT, alanine aminotransferase; PLT, platelet; PSM, propensity score matching; RFA, radiofrequency ablation; RHR, repeat hepatic resection; TBIL, total bilirubin; WBC, white blood cell.

**Table 2:** Baseline characteristics of primary HCC before and after PSM

Variables	Before matching			After matching		
	RFA (n = 194)	RHR (n = 138)	*P*	RFA (n = 120)	RHR (n = 120)	*P*
AFP, ng/mL						
>20	116 (59.8)	84 (60.9)	.844	67 (55.8)	73 (60.8)	.432
≤20	78 (40.2)	54 (39.1)		53 (44.2)	47 (39.2)	
Tumor size, cm	5.0 ± 2.7	4.6 ± 2.3	.132	5.1 ± 2.8	4.4 ± 2.3	.058
Tumor number						
Solitary	168 (86.6)	123 (89.1)	.489	106 (88.3)	106 (88.3)	1.000
Multiple	26 (13.4)	15 (10.9)		14 (11.7)	14 (11.7)	
Tumor capsule						
Complete	78 (40.2)	60 (43.5)	.551	51 (42.5)	51 (42.5)	1.000
Incomplete	116 (59.8)	78 (56.5)		69 (57.5)	69 (57.5)	
Tumor extent						
Unilobar	184 (94.8)	131 (94.9)	.973	117 (97.5)	113 (94.2)	.196
Bilobar	10 (5.2)	7 (5.1)		3 (2.5)	7 (5.8)	
Microvessel invasion of the initial tumor						
Present	39 (20.1)	26 (18.8)	.775	22 (18.3)	23 (19.2)	.869
Absent	155 (79.9)	112 (81.2)		98 (81.7)	97 (80.8)	
Histology						
Well	20 (10.3)	17 (12.3)	.566	20 (16.7)	17 (14.2)	.592
Poorly and moderately	174 (89.7)	121 (87.7)		100 (83.3)	103 (85.8)	
Milan criteria						
Within	104 (53.6)	86 (62.3)	.114	66 (55.0)	75 (62.5)	.238
Beyond	90 (46.4)	52 (37.7)		54 (45.0)	45 (37.5)	
TNM stage						
Ⅰ	138 (71.1)	100 (72.5)	.791	90 (75.0)	85 (70.8)	.468
Ⅱ‐ⅢA	56 (28.9)	38 (27.5)		30 (25.0)	35 (29.2)	
Surgical margin, cm	1.2 ± 0.9	1.2 ± 0.8	.982	1.2 ± 0.9	1.2 ± 0.9	.941
Time of Pringle's maneuver, min	13.3 ± 11.2	11.9 ± 9.3	.232	13.4 ± 11.2	11.9 ± 9.4	.270
Blood loss, mL						
>200	88 (45.4)	57 (41.3)	.463	58 (48.3)	51 (42.5)	.364
≤200	106 (54.6)	81 (58.7)		62 (51.7)	69 (57.5)	
Extent of liver resection^a^						
Major	29 (14.9)	26 (18.8)	.347	15 (12.5)	24 (20.0)	.115
Minor	165 (85.1)	112 (81.2)		105 (87.5)	96 (80.0)	

Note

Variables are expressed as the mean ± SD or n (%)

Abbreviations: AFP, α‐fetoprotein; HCC, hepatocellular carcinoma; PSM, propensity score matching; RFA, radiofrequency ablation; RHR, repeat hepatic resection; TNM, tumor‐node‐metastasis.

^a^ Major liver resection: resection of three or more segments; minor liver resection: resection of fewer than three segments.aaa

### 3.1 Postrecurrence survival

**Figure 1:**
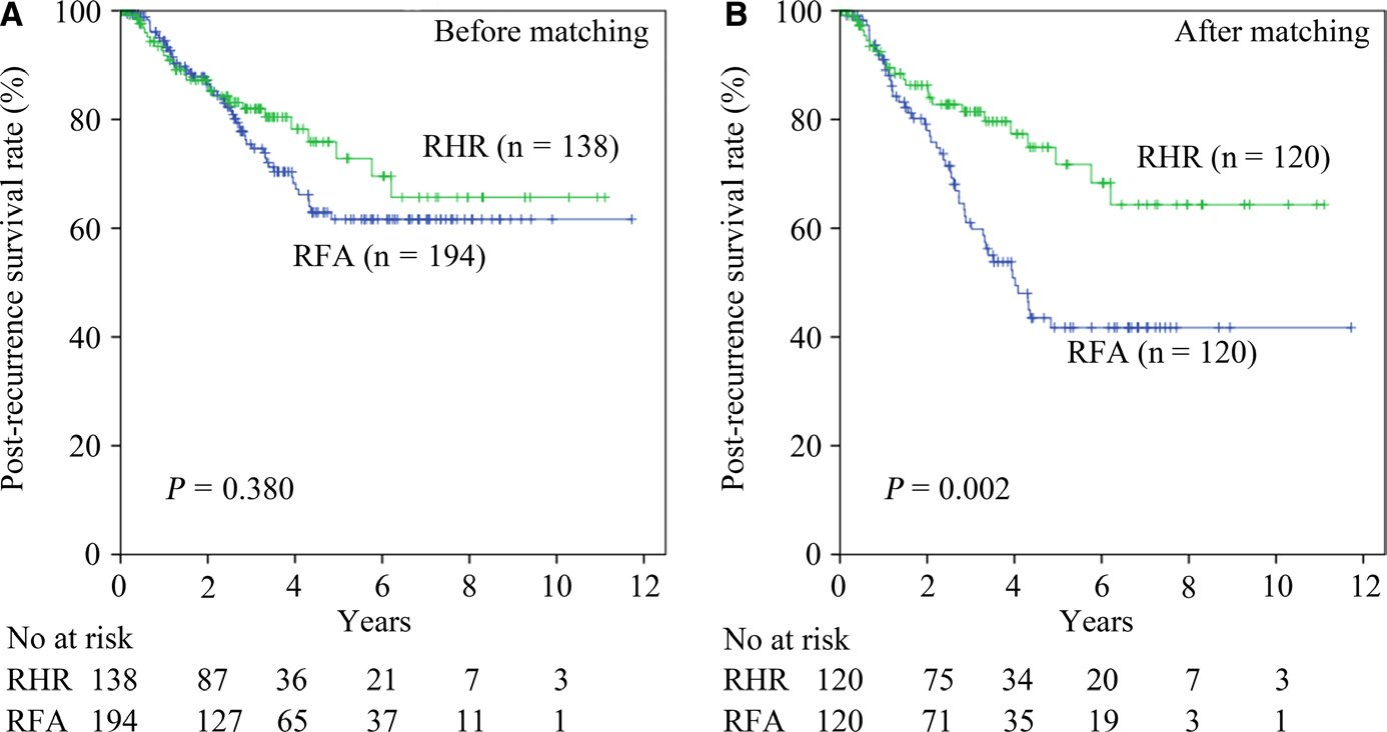
The postrecurrence survival rates of recurrent hepatocellular carcinoma patients in the repeat hepatic resection (RHR) group compared with the radiofrequency ablation (RFA) group before (A) and after (B) propensity score matching analysis

Before matching, a total of 78 patients died from recurrent HCC, including 52 of the 194 (26.8%) patients in the RFA cohort and 26 of the 138 (18.8%) patients in the RHR cohort. For the RHR group, the 1‐, 3‐, and 5‐year PRS rates were 91.8%, 82.0%, and 72.9%, respectively. In contrast, for the RFA group, the 1‐, 3‐, and 5‐year PRS rates were 94.4%, 75.4%, and 61.7%, respectively (*P* = .380) (Figure 1A). After matching, a total of 76 patients died from recurrent HCC, including 52 of 120 (43.3%) patients in the RFA cohort and 24 of 120 (20.0%) patients in the RHR cohort. For the RHR group, the 1‐, 3‐, and 5‐year PRS rates were 90.5%, 81.5%, and 71.8%, respectively. For the RFA group, the respective 1‐, 3‐, and 5‐year PRS rates were 91.0%, 61.0%, and 41.7%, respectively (*P* = .002) (Figure 1B).

### 3.2 Subgroup analysis of PRS

**Figure 2:**
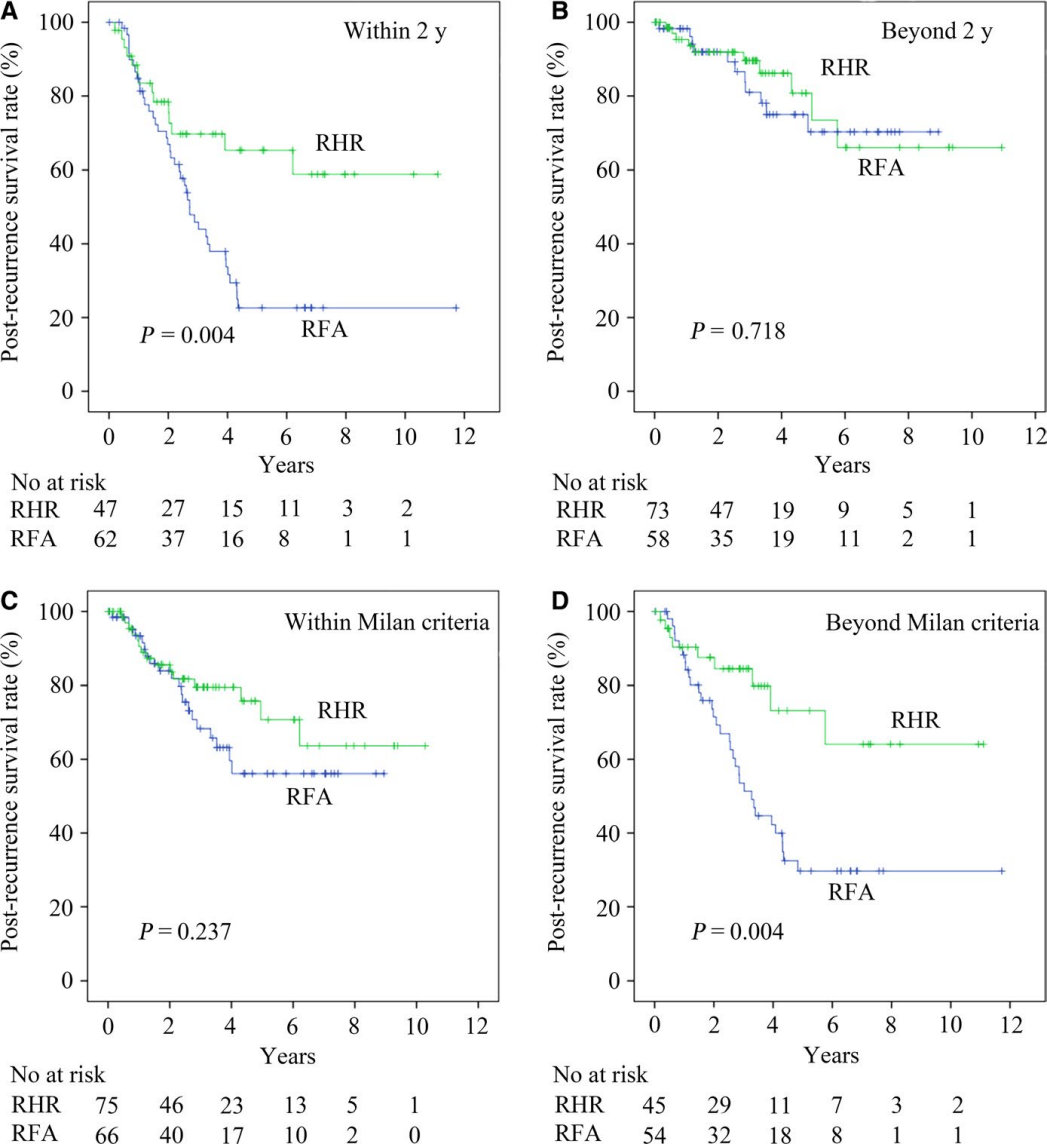
Subgroup analysis of the postrecurrence survival rates of recurrent hepatocellular carcinoma patients in the repeat hepatic resection (RHR) group compared with the radiofrequency ablation (RFA) group: patients who relapsed (A) within 2 y and (B) beyond 2 y after initial hepatic resection; patients whose initial tumor burden (C) was within and (D) beyond the Milan criteria

For 47 patients in the RHR group and 62 patients in the RFA group who presented tumor recurrence within 2 years after the initial hepatic resection, the respective 1‐, 3‐, and 5‐year PRS rates were 83.5%, 69.7%, and 65.4% for the RHR group and 84.8%, 45.9%, and 22.7%, respectively, for the RFA group (*P* = .004) (Figure 2A). For patients with an interval of tumor recurrence >2 years, there was no significant difference in the PRS rates between patients treated by RHR or RFA (*P* = .718) (Figure 2B).

For 45 patients in the RHR group and 54 patients in the RFA group who had an initial tumor burden beyond the Milan criteria, the respective 1‐, 3‐, and 5‐year PRS rates were 90.4%, 84.5%, and 64% in the RHR group and 88.2%, 53.6%, and 29.8%, respectively, in the RFA group (*P* = .004) (Figure 2D). There was no significant difference in PRS rates between patients with an initial tumor burden within the Milan criteria (*P* = .237) (Figure 2C).

### 3.3 Univariate and multivariate analyses of PRS

With regard to PRS, age > 55 years (hazard ratio [HR]: 1.60, 95% confidence interval [CI]: 1.00‐2.53, *P* = .048), the presence of hepatitis (HR: 2.78, 95% CI: 1.41‐5.26, *P* = .003), time to recurrence >2 years (HR: 0.31, 95% CI: 0.19‐0.51, *P* < .001), serum AFP level at recurrence >20 ng/mL (HR: 2.04, 95% CI: 1.30‐3.21, *P* = .002), and treatment allocation (HR: 0.47, 95% CI: 0.29‐0.77, *P* = .002) were identified as significant factors in the univariate analysis. In the multivariate analysis using the Cox proportional hazard model, age > 55 years (HR: 1.78, 95% CI: 1.11‐2.88, *P* = .018), the presence of hepatitis (HR: 2.27, 95% CI: 1.14‐4.55, *P* = .020), time to recurrence >2 years (HR: 0.34, 95% CI: 0.21‐0.58, *P* < .001), serum AFP level at recurrence >20 ng/mL (HR: 1.79, 95% CI: 1.13‐2.84, *P* = .013), and treatment allocation (HR: 0.54, 95% CI: 0.33‐0.88, *P* = .014) were evaluated as significant factors (Table 3).

**Table 3:** Univariate and multivariate analyses of prognostic factors for PRS

Variables	Univariate			Multivariate		
	HR	95% CI	*P*	HR	95% CI	*P*
Characteristics of primary HCC						
AFP (>/≤20 ng/mL)	1.39	0.87‐2.22	.165			
Tumor size (>/≤3 cm)	1.20	0.72‐2.00	.481			
Tumor number (>/≤1)	1.54	0.84‐2.79	.160			
Tumor capsule (yes/no)	0.93	0.59‐1.47	.765			
Tumor extent (unilobar/bilobar)	0.87	0.27‐2.75	.806			
Microvessel invasion of the initial tumor (yes/no)	1.62	0.89‐2.95	.116			
Histology (well/poor and moderate)	0.62	0.33‐1.15	.127			
Surgical margin (>/≤1 cm)	1.05	0.66‐11.67	.833			
Time of Pringle's maneuver (>/≤20 min)	1.06	0.63‐1.79	.818			
Characteristics at the time of recurrence						
Age (>/≤55 y)	1.60	1.00‐2.53	.048	1.78	1.11‐2.88	.018
Etiology (virus/others)	2.78	1.41‐5.26	.003	2.27	1.14‐4.55	.020
PLT (>/≤100×10^9^/L)	0.80	0.44‐1.46	.467			
ALB (>/≤35 g/L)	0.71	0.22‐2.26	.564			
TBIL (>/≤17 mmol/L)	1.27	0.78‐2.04	.334			
Cirrhosis (yes/no)	1.04	0.64‐1.71	.868			
Time to recurrence (>/≤2 y)	0.31	0.19‐0.51	<.001	0.34	0.21‐0.58	<.001
Tumor size at recurrence (>/≤3 cm)	0.97	0.52‐1.80	.922			
Tumor number at recurrence (>/≤1)	1.43	0.85‐2.40	.181			
AFP at recurrence (>/≤20 ng/mL)	2.04	1.30‐3.21	.002	1.79	1.13‐2.84	.013
Treatment allocation (RHR/RFA)	0.47	0.29‐0.77	.002	0.54	0.33‐0.88	.014

Abbreviations: AFP, α‐fetoprotein; ALB, albumin; CI, confidence interval; HR, hazard ratio; PLT, platelet; PRS, postrecurrence survival; RFA, radiofrequency ablation; RHR, repeat hepatic resection; TBIL, total bilirubin.

### 3.4 Treatment complications

Treatment‐related complications are summarized in Table 4. Complications were reported according to the Clavien‐Dindo grade.^22^ Major complications were those classified as Clavien‐Dindo grade III or higher. No treatment‐related mortality was reported in this study, and the incidence of major complications did not differ significantly between the RHR and RFA groups (Table 4).

**Table 4:** Major complications after RHR and RFA

Variables	RFA (n = 120)	RHR (n = 120)	*P*
Postoperative hemorrhage			
Absent	120	118	.478
Present	0	2	
Ascites			
Absent	115	115	1.000
Present	5	5	
Bile leakage			
Absent	120	120	1.000
Present	0	0	
Pleural effusion			
Absent	120	119	1.000
Present	0	1	
Liver failure			
Absent	120	118	.478
Present	0	2	

Note

Major complications were defined as those with a Clavien‐Dindo classification of grade III or higher.

Abbreviations: RFA, radiofrequency ablation; RHR, repeat hepatic resection.

## 4 DISCUSSION

This study demonstrated that RHR was more effective than RFA for extending the postoperative recurrence survival time in HCC patients after the initial recurrence, especially for patients who relapsed within 2 years or those who had an initial tumor burden beyond the Milan criteria.

The Barcelona clinic liver cancer (BCLC) staging system was initially established as a link between staging and treatment indications for primary HCC.^23^ For naïve HCC patients, it is generally accepted that the survival outcome of RFA is comparable to hepatic resection for small HCC.^24^, ^25^, ^26^ However, the treatment of recurrent HCC does not strictly follow the BCLC staging system, as expected differences should be considered when determining treatment options for recurrent tumors. First, the size of the recurrent tumor may be smaller than the initial tumor due to more frequent surveillance. Because all patients enrolled in this study were diagnosed with intrahepatic recurrent HCC after the initial curative hepatic resection, the finding that the median tumor size of the primary HCC summarized in the table was >5 cm is reliable, since RFA may be inferior for local tumor control. However, due to more frequent surveillance after initial resection, the median size of recurrent HCC in both cohorts in this study was mostly <3 cm, with no significant difference observed, as summarized in Table 1 (RHR vs RFA: 2.4 ± 1.1 cm vs 2.2 ± 1.0 cm, *P* = .091). In addition, the limitations in liver function reserve cannot be ignored after the initial resection. Several studies reported generally comparable outcomes between RHR and RFA,^13^, ^14^, ^15^, ^16^ although a tendency toward longer survival time was observed in the RHR group than in the RFA group. Conversely, others have argued that RHR should be considered the most effective treatment for intrahepatic recurrence HCC.^7^, ^9^, ^11^ In summary, the study limitations, including the small sample size and short follow‐up period, may have led to our failure to identify an association between PRS and risk factors.

In addition to the type of treatment, our study identified the presence of hepatitis and serum AFP level at recurrence as independent factors associated with PRS in the multivariate analysis. Among patients who relapse within 2 years or who have an initial tumor burden beyond the Milan criteria, RHR provided a survival advantage for recurrent HCC compared with RFA. As HCC is more likely to microdisseminate into the tributaries of the portal branches and shed tumor emboli into the neighboring branches of the same liver segment,^27^ RHR may offer a better chance to eradicate intrahepatic micrometastases generated from the original tumor than RFA. Another explanation may be that RFA was reported to be inferior to surgery for local tumor control.^24^, ^25^ Relapse beyond 2 years after initial resection is now generally believed to be due to multicentric carcinogenesis because of hepatitis or cirrhosis,^28^, ^29^ and our study showed that there was no significant difference in the long‐term outcomes of patients who received RHR or RFA, consistent with previous studies.^30^, ^31^, ^32^ In fact, the rate of repeated resection in clinical practice was less than 30%.^33^ In addition to the limited liver remnant, another possible obstacle to RHR may be the assumption that RHR is more difficult to perform due to postoperative adhesion and anatomic changes. However, a systematic review reported that the 5‐year patient survival rate after recurrence who underwent repeated liver resection ranged from 22.0% to 84.0%,^34^ and the data of Yoh et al showed that the 5‐year survival rate after recurrence was 73.8% for HCC patients with intrahepatic recurrence who underwent repeat resection,^35^ which was consistent with our results. Thus, the results of our study indicated that RHR should not be ignored but performed carefully when surgical resection is considered feasible in clinical practice. Although RFA is generally regarded as safer and less invasive than RHR, both treatment groups suffered similar treatment complication morbidity rates. This may be related to the introduction of PSM and the improvement in the perioperative management of modern surgery.

The limitations of this study should also be noted. First, deviation may be unavoidable due to the retrospective design of our study. Second, treatment selection for recurrent HCC was decided by our multidisciplinary team and not randomly assigned. For HCC patients who had major resection, when recurrence was present and located deep within the liver or in a patient with insufficient liver function reserve, RHR should be carefully considered to determine whether it would offer any benefit to the patient. Thus, a prospective, randomized trial is needed to compare the efficacy of RHR and RFA for the treatment of recurrent HCC.

In conclusion, the results of our study showed that RHR is relatively safe and yielded better PRS rates than RFA for recurrent HCC, especially in patients who relapse within 2 years or who have a primary tumor burden beyond the Milan criteria.

## AUTHOR CONTRIBUTIONS

Liang‐He Lu: Conceptualization, project administration, formal analysis, software, and writing—review and editing. Jie Mei: Conceptualization, formal analysis, software, and writing‐original draft. Anna Kan: Data curation, formal analysis, methodology. Shao‐Hua Li: Data curation, formal analysis, funding acquisition. Wei Wei: Data curation, formal analysis, funding acquisition. Mu‐Yan Cai: Data curation, formal analysis, resource. Min‐Shan Chen: Data curation, project administration, resources. Yong‐Fa Zhang: Conceptualization, software, writing—original draft. Rong‐Ping Guo: Conceptualization, supervision, writing—review and editing.

## Data Availability

Key raw data of this study have been uploaded onto the Research Data Deposit public platform (http://www.researchdata.org.cn) (no. RDDA2019001076).
